# Cardiovascular disease and multimorbidity: A call for interdisciplinary research and personalized cardiovascular care

**DOI:** 10.1371/journal.pmed.1002545

**Published:** 2018-03-27

**Authors:** Kazem Rahimi, Carolyn S. P. Lam, Steven Steinhubl

**Affiliations:** 1 University of Oxford, Oxford, United Kingdom; 2 National Heart Centre Singapore, Singapore, Singapore; 3 Scripps Translational Science Institute, La Jolla, California, United States of America

## Abstract

In a Guest Editorial, Kazem Rahimi, Carolyn Lam, and Steven Steinhubl call for interdisciplinary research and personalized cardiovascular care to better manage patients with cardiovascular disease and multimorbidity.

Advances in medicine and population health over the past few decades have led to an unprecedented increase in life expectancy. Today, for the first time in history, we have reached a point at which more people can expect to live into their 60s, and beyond, than to die before the age of 60. However, because mortality has been declining faster than disease incidence and disability, many people who are now reaching advanced ages are likely to experience multimorbidity, commonly defined as the presence of 2 or more chronic medical conditions in an individual. As a group of conditions, cardiovascular disease (CVD) exemplifies several challenges of multimorbidity. Although the typical 21st-century CVD patient is older than 65 years and has multiple diseases, most clinical research is still focused on management of single risk factors or diseases in isolation. This leaves several major gaps in our knowledge about disease–disease interactions and how these might affect disease occurrence, treatment patterns, and outcomes. It is in this context that *PLOS Medicine* is devoting this Special Issue to research and discussion focused on multimorbidity and CVD.

Several important studies in this issue shed light on the burden, patterns, and outcomes of multimorbidity in patients with CVD. In a large-scale population-based study, Jenny Tran and colleagues investigated the prevalence of 56 major chronic conditions prior to diagnosis of incident nonfatal ischemic heart disease (IHD) or stroke [1] and show that, although the age/sex-standardized incidence of IHD and stroke fell by 34% during 2000–2014, the proportion of CVD patients with higher numbers of comorbidities increased substantially—a trend that was not due to population ageing. Even in age/sex-standardized models, the proportion of patients with 5 or more comorbidities increased 4-fold from 6.3% to 24.3%. In another large-scale study, Marlous Hall and colleagues confirm the large burden of multimorbidity in a population of almost 700,000 patients who presented with acute myocardial infarction [2] and show a graded negative association between higher burden of multimorbidity and receipt of pharmacological treatment for secondary prevention of CVD. They further show that patients with a higher burden of multimorbidity were at substantially increased risk of death several years after the acute event.

The importance of noncardiovascular comorbidities is highlighted in several studies in the issue. In the population-based study by Tran et al. [1], comorbidities considered “discordant” with CVD (i.e., not perceived to share pathophysiological pathways, such as arthritis and mental illness) constituted 4 of the 10 most common comorbidities—a high prevalence that suggests a potential role of common etiologies or shared risk factors (e.g., social stress and inflammation) in determining the trajectory of multimorbidity in CVD. Davide Vetrano and colleagues [3] similarly show that the presence of neuropsychiatric multimorbidity was associated with declines in both mobility and independence in older adults, whereas isolated cardiovascular multimorbidity was only associated with loss of physical mobility.

Patients with heart failure represent a group in which virtually all individuals have multimorbidity, and the impact of multimorbidities on the trajectory of heart failure is increasingly appreciated. Claire Lawson and colleagues aimed to elucidate how different comorbidities may impact on quality of life and health among 10,575 patients from the Swedish Heart Failure Registry [4]. They found that noncardiovascular comorbidities were associated with a higher overall symptom burden and more severe symptoms than cardiovascular comorbidities. Furthermore, cardiovascular comorbidities were more likely to be associated with pain and anxiety than shortness of breath or fatigue. Jasper Tromp et al. applied latent class analysis to 6,480 patients with heart failure from the Asian Sudden Cardiac Death in Heart Failure (ASIAN-HF) registry [5] and found that comorbidities clustered into 5 distinct patterns, each differentially impacting patients’ quality of life and clinical outcomes. These papers underscore the heterogeneity and importance of multimorbidity in heart failure. Given that current guidelines focus on cardiovascular status and common symptoms in heart failure, these findings also suggest that, to improve outcomes, individualized person-centered care targeting specific comorbidities and associated symptoms may be warranted.

What do we know about factors that could drive accumulation of multimorbidity? Xiaolin Xu and colleagues investigated the clustering of cardiometabolic conditions in a cohort of middle-aged women without diabetes, heart disease, or stroke at baseline [6] and report that the probability of developing a second or third cardiometabolic condition was significantly higher than the probability of a transition from no disease to a single cardiometabolic disease. This pattern of disease clustering was explained by several established risk factors at baseline and their accumulation over time. This is broadly consistent with Gloria Aguayo and colleagues’ study comparing the association between 35 previously published multivariable frailty scores and risk of incident CVD, incident cancer, and all-cause mortality [7]. Although 28 out of 35 frailty scores were found to have significant added predictive value for all-cause mortality, none improved basic prediction models for CVD or cancer. But could novel biomarkers still enhance our ability to predict who is more likely to suffer an adverse outcome and enable more targeted approaches to risk management? Insights into this question come from 2 studies in the issue. Evan Muse and colleagues show that a genetic score was able to predict incident atrial fibrillation independent of established clinical risk factors [8], alongside Mandip Dhamoon and colleagues’ observation of a predictive role for cerebral white matter hyperdensities in increased risk of decline in functional status over the long term, independent of conventional risk factors [9].

The major challenge facing clinicians is how to translate a vast amount of population-based information into the best care for their patient, which is especially challenging in the setting of multimorbidity. An important contribution to better understanding the value of tailoring management to the individual comes from the work of Kunal Karmali et al., who used participant-level data from nearly 50,000 individuals with high blood pressure (BP) to show that assessment of an individual’s CVD risk, rather than a BP cutoff, to guide treatment would lead to overall better outcomes while treating fewer people [10]. Treating high BP in individuals with a significant comorbidity such as HIV infection offers both challenges and opportunities, especially in a low-resource setting, and Pragna Patel and team describe the experience of creating a scalable system of care in Malawi that can effectively address both HIV and noncommunicable diseases [11]. One of the great challenges to implementing more individualized care is the enormous amount of data available and the need for any clinician to be able to expertly synthesize it all. Automated clinical decision support (CDS) can help overcome this data overload, and the cluster-randomized trial done by Lars Karlsson and colleagues demonstrates both the potential and challenges of incorporating CDS into clinical practice [12]. These studies highlight that we are at the cusp of nearly unimaginable data-driven changes in healthcare that will enable more, and more precise, individualized and patient-centric care.

Collectively, the breadth and depth of the original research and associated perspectives in this Special Issue of *PLOS Medicine* make a substantial contribution towards our understanding of multimorbidity in CVD. At the same time, they highlight many priorities and opportunities for research, showing that understanding multimordibity requires dealing with complexity and understanding patients’ experiences, emphasizing the importance of interdisciplinary research. We welcome and look forward to such research in the future.

## Abbreviations

ASIAN-HF: Asian Sudden Cardiac Death in Heart Failure
BP: blood pressure
CDS: clinical decision support
CVD: cardiovascular disease
IHD: ischemic heart disease
